# 173. Identifying Opportunities for Urine Culture Diagnostic Stewardship in a Pediatric Health System

**DOI:** 10.1093/ofid/ofae631.053

**Published:** 2025-01-29

**Authors:** Hee-won Yoon, Didien Meyahnwi, Kathleen Chiotos, Michelle Dunn, Rebecca Green, Brandon Ku, Charmaine Mutucumarana, Maya Overland, Tracey Polsky, Sharer Gwyneth, Kenneth Smith, Sanjeev K Swami, Rebecca Same

**Affiliations:** Children's Hospital of Philadelphia, Philadelphia, Pennsylvania; Children's Hospital of Philadelphia, Philadelphia, Pennsylvania; Children's Hospital of Philadelphia, Philadelphia, Pennsylvania; Children's Hospital of Philadelphia, Philadelphia, Pennsylvania; Children's Hospital of Philadelphia, Philadelphia, Pennsylvania; Children's Hospital of Philadelphia, Philadelphia, Pennsylvania; Children's Hospital of Philadelphia, Philadelphia, Pennsylvania; Children's Hospital of Philadelphia, Philadelphia, Pennsylvania; Children's Hospital of Philadelphia, Philadelphia, Pennsylvania; Children's Hospital of Philadelphia, Philadelphia, Pennsylvania; Children's Hospital of Philadelphia, Philadelphia, Pennsylvania; Children's Hospital of Philadelphia, Philadelphia, Pennsylvania; Children's Hospital of Philadelphia, Philadelphia, Pennsylvania

## Abstract

**Background:**

Reflex urine cultures based on urinalysis (UA) parameters are commonly used to avoid excess urine cultures and reduce treatment of asymptomatic bacteriuria in adults. Reflex testing is less common in pediatrics and optimal UA parameters in children are unknown. We aimed to evaluate performance of laboratory and point of care (POC) UA for prediction of significant bacteriuria in children.

**Figure:**
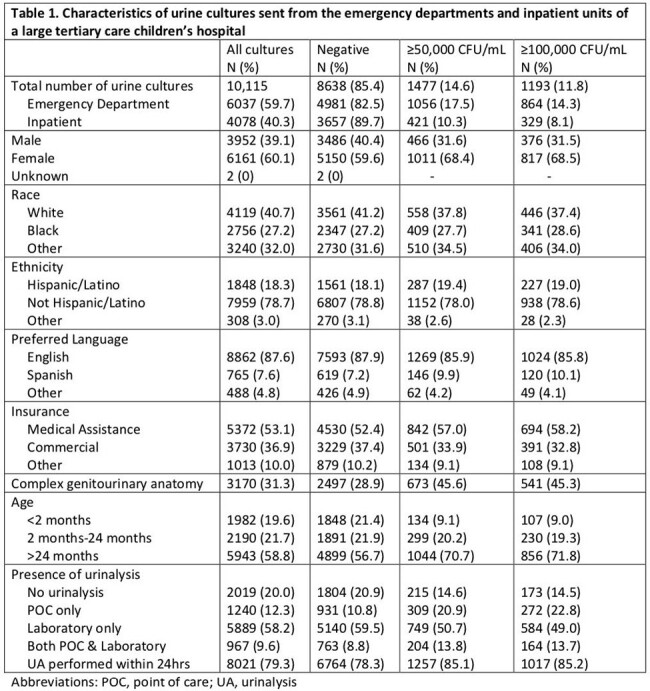

**Methods:**

We performed a retrospective cohort study of all urine cultures performed in the emergency departments and inpatient units of two hospitals within a tertiary care children’s health system between January 2023 and March 2024. We identified core laboratory-performed UAs (macroscopic UA with reflex to microscopic UA for presence of blood, leukocyte esterase (LE), nitrite, or protein) and POC UAs performed within 48 hours of each culture to characterize diagnostic practices. We defined a negative culture as < 50,000 CFU/mL of bacteria or results consistent with contamination. Limiting analysis to UAs performed within 24 hours of a culture, we then determined the performance characteristics of POC and lab UA parameters for prediction of significant bacteriuria, using a threshold of ≥ 50,000 CFU/mL and ≥ 100,000 CFU/mL.

**Figure:**
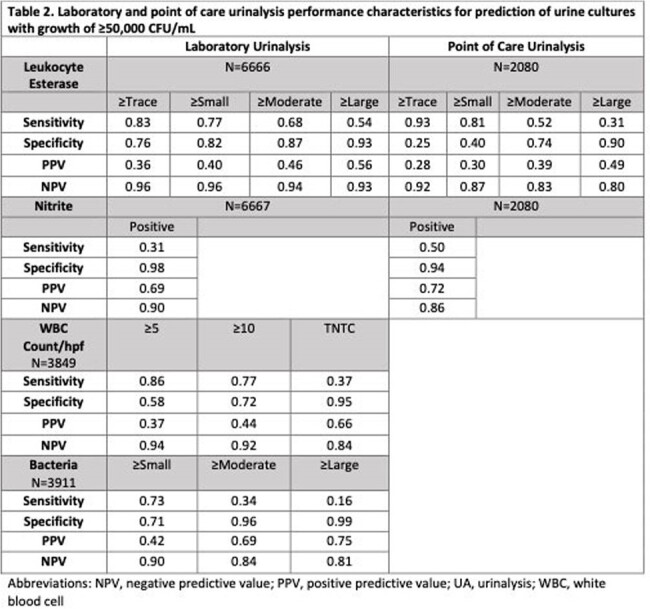

**Results:**

There were a total of 10,115 urine cultures performed in 7,152 children (Table 1). There were 8,638 (85%) negative cultures. No UA was done in 2,019 (20%). Of cultures with a UA, 8,021 (99%) were done within 24 hours. Of 6,856 cultures with a lab UA, 4,828 (70%) had < 5 WBCs/hpf or no WBC reported due to negative macroscopic UA. Of these, 4,608 (95%) cultures were negative. At least 5 WBCs/hpf had a sensitivity of 86%, specificity of 55%, and negative predictive value (NPV) of 93.5% for growth of ≥ 50,000 CFU/mL in culture (Table 2). POC UA LE had similar sensitivity but lower specificity, positive predictive value (PPV), and NPV compared to lab UA. POC UA nitrite had higher sensitivity and similar specificity, PPV, and NPV compared to lab UA (Table 3).

**Figure:**
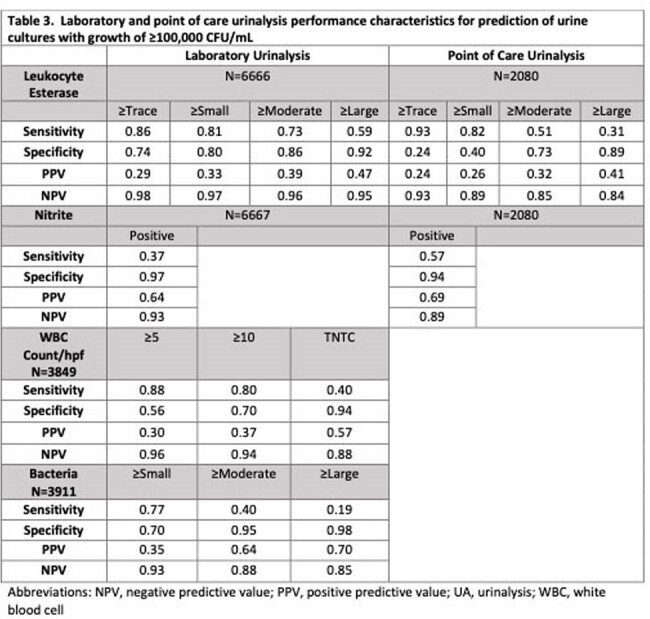

**Conclusion:**

Over 10,000 urine cultures were sent in a 15-month period. Pyuria had a high NPV for significant bacteriuria. Because 70% of lab UAs do not have pyuria, implementation of a reflex urine culture could eliminate many unnecessary urine cultures without significantly compromising detection of bacteriuria.

**Disclosures:**

**All Authors**: No reported disclosures

